# Establishment of Trophoblast Stem Cells under Defined Culture Conditions in Mice

**DOI:** 10.1371/journal.pone.0107308

**Published:** 2014-09-09

**Authors:** Yasuhide Ohinata, Tomoyuki Tsukiyama

**Affiliations:** 1 PRESTO, Japan Science and Technology Agency, Kawaguchi, Saitama, Japan; 2 Life Science Experimental Facility, Department of Biotechnology, Faculty of Life and Environmental Sciences, University of Yamanashi, Kofu, Yamanashi, Japan; 3 Laboratory for Pluripotent Stem Cell Studies, Center for Developmental Biology, RIKEN Kobe institute, Kobe Chuo-ku, Hyogo, Japan; Michigan State University, United States of America

## Abstract

The inner cell mass (ICM) and trophoblast cell lineages duet early embryonic development in mammals. After implantation, the ICM forms the embryo proper as well as some extraembryonic tissues, whereas the trophoectoderm (TE) exclusively forms the fetal portion of the placenta and the trophoblast giant cells. Although embryonic stem (ES) cells can be derived from ICM in cultures of mouse blastocysts in the presence of LIF and/or combinations of small-molecule chemical compounds, and the undifferentiated pluripotent state can be stably maintained without use of serum and feeder cells, defined culture conditions for derivation and maintenance of undifferentiated trophoblast stem (TS) cells have not been established. Here, we report that addition of FGF2, activin A, XAV939, and Y27632 are necessary and sufficient for derivation of TS cells from both of E3.5 blastocysts and E6.5 early postimplantation extraembryonic ectoderm. Moreover, the undifferentiated TS cell state can be stably maintained in chemically defined culture conditions. Cells derived in this manner expressed TS cell marker genes, including *Eomes*, *Elf5*, *Cdx2, Klf5, Cdh1, Esrrb, Sox2*, and *Tcfap2c*; differentiated into all trophoblast subtypes (trophoblast giant cells, spongiotrophoblast, and labyrinthine trophoblast) *in vitro*; and exclusively contributed to trophoblast lineages in chimeric animals. This delineation of minimal requirements for derivation and self-renewal provides a defined platform for precise description and dissection of the molecular state of TS cells.

## Introduction

The placenta plays essential roles in mammalian embryonic development. This vital organ serves as a point of contact between the fetal and maternal circulation, allowing transfer of nutrients and oxygen to the fetus and metabolic waste back to the mother. In addition to its role as an interface between the developing embryo and its mother, the placenta also produces hormones that play a variety of roles during pregnancy and provides the fetus with immune protection. The primary components of the placenta are derived from the trophoblast lineage, a single layer of cells within the blastocyst that surrounds the inner cell mass (ICM), from which the embryo is ultimately derived [1]. Over the past decades, ICM-derived embryonic stem (ES) cells and trophoectoderm (TE)-derived trophoblast stem (TS) cells have substantially advanced our understanding of the molecular mechanisms of early embryonic and placental development. ES cells were first manipulated and characterized in the early 1980s [2], [3]. The conditions for derivation and culture of ES cells in the laboratory, based on a large body of experimental work, include a wide variety of components, including non-dividing “feeder cells” that provide growth factors and extracellular matrix support; conditioned media from other cell types, which also provide soluble factors; serum; and individual empirically determined factors such as growth factors, cytokines, and hormones [2]–[7]. Currently, as a result of extensive trial and error, undifferentiated pluripotent stem cells can efficiently be derived and maintained under chemically defined culture conditions in the presence of leukemia inhibitory factor (LIF) and/or small-molecule chemical compounds (i.e., PD0325901, PD184352, PD173074, SU5402, CHIR99021, etc.) [8], [9]. On the other hand, since TS cells were first described [10], they have been derived and maintained using various combinations of primary mouse embryonic fibroblast (MEF) cells, conditioned media, fetal bovine serum (FBS), fibroblast growth factor 4 (FGF4), and heparin. Recently, Kubaczka et al. reported derivation and maintenance of TS cells using a chemically defined medium containing FGF4, heparin, and TGFβ1 on Matrigel [11]. Here we report that fibroblast growth factor 2 (FGF2), activin A, and the small molecules XAV939 and Y27632 in chemically defined medium (CDM/FAXY) on fibronectin are sufficient for derivation and maintenance of TS cells from both E3.5 TE and E6.5 early-postimplantation extraembryonic ectoderm (ExE). XAV939 is a canonical Wnt signaling inhibitor, which stimulates β-catenin degradation by stabilizing axin via inhibition of poly-ADP-ribosylating enzymes tankyrase 1 and tankyrase 2 [12], and Y27632 is an inhibitor of the Rho-associated protein kinase p160ROCK [13]. The TS cells that grew under these conditions satisfied all the criteria for undifferentiated TS cells: self-renewal capacity, marker gene expression, differentiation competence *in vitro*, and ability to contribute to the placenta in chimeric mice.

## Results

### Establishment and self-renewal of TS cells in chemically defined conditions

**Figure 1:**
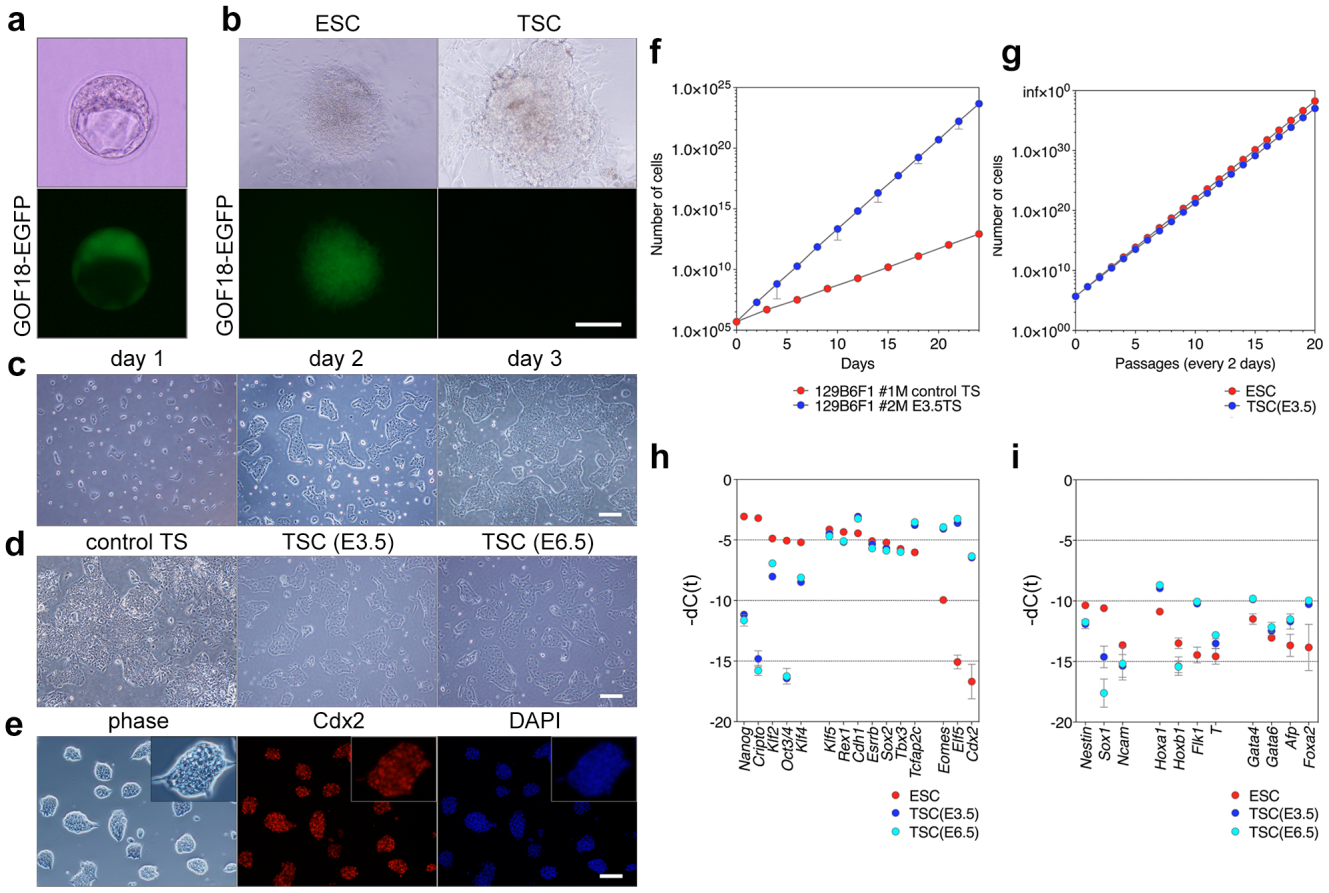
Derivation of TS cells in CDM/FAXY. (a) E3.5 blastocyst with the GOF18-EGFP (*Oct3/4*-EGFP) reporter transgene. Scale bar, 50 µm. (b) Outgrowths arising at day 5 in CDM/LIF/2i (left) and CDM/FAXY (FGF2, 12.5 ng/ml) (right) on fibronectin. Scale bar, 100 µm. (c) Morphology of TS cell colonies at day 1 (left), day 2 (center), and day 3 (right) after passage. Scale bar, 100 µm. (d) Derived TS cells from E3.5 blastocyst (center) and E6.5 ExE (right). Conventional TS cells from an E3.5 blastocyst (left) are shown as a control. Scale bar, 100 µm. (e) Immunofluorescence staining of TS cells on a fibronectin-coated plastic-bottom dish. Scale bar, 100 µm. (f) Proliferation of conventional TS cells (red) and E3.5 blastocyst–derived TS cells in CDM/FAXY (blue). (g) Proliferation of ES cells in CDM/LIF/2i (red) and E3.5 blastocyst–derived TS cells in CDM/FAXY (blue). (h) Comparison of expression levels of ES cell–specific genes, commonly expressed genes, and TS cell–specific genes among ES cells (red), E3.5 blastocyst–derived TS cells (blue), and E6.5 ExE–derived TS cells (cyan). (i) Comparison of expression levels of ectodermal genes, mesodermal genes, and endodermal genes among ES cells (red), E3.5 blastocyst–derived TS cells (blue), and E6.5 ExE–derived TS cells (cyan).

To observe the responses of blastocysts to activation of major signaling pathway (e.g., BMP, activin, TGFβ, FGF, WNT, etc.), we initially cultured mouse blastocysts in chemically defined medium (CDM) containing various combinations of BMP4, BMP7, LDN-193189, Dorsomorphin, activin A, TGFβ1, GDF3, SB431542, A-83-01, LIF, FGF2, FGF4, PD0325901, PD184352, PD173074, SU5402, WNT3, CHIR99021, XAV939, GF109203X, and Y27632. The results showed that the combination of FGF2, activin A, XAV939, and Y27632 in CDM gave rise to rapid TE proliferation from E3.5 blastocysts. To this end, we cultured CD1 × B6 F_1_ GOF18 blastocysts harboring the *Oct3/4-EGFP* reporter (Fig. 1a) [14] in CDM containing LIF, PD0325901 (a MEK inhibitor), and CHIR99021 (a GSK3 inhibitor) (CDM/L2i, the ES cell culture condition) or CDM containing FAXY (12.5 ng/ml FGF2, 20 ng/ml activin A, 10 nM XAV939, 5 nM Y27632) (CDM/FAXY, the TS cell culture condition). After 5 days, inner cell masses (ICM) grown in CDM/L2i gave rise to *Oct3/4-EGFP*–positive outgrowths (Fig. 1b, left). By contrast, the outer layer of blastocysts (trophoectoderm, TE), grown in CDM/FAXY formed *Oct3/4-EGFP*–negative outgrowths (Fig 1b, right). These primary TE explants could be propagated by single-cell dissociation according to the protocol used to passage mouse ESCs at 2–3-day intervals. Passaged cells initially formed compact and domed colonies, and then gradually changed shape to assume the characteristic morphology of TS cells (Fig. 1c). Next, we collected morulas and blastocysts (CD1 × B6 F_1_, CD1 × CD1, or 129 × B6 F_1_) and cultured them in CDM containing various combinations of FGF2, FGF4, activin A, XAV939, and Y27632. In all genetic backgrounds, TS cell lines could only be efficiently derived in CDM containing FAXY (Table. 1). In CD1 × B6 F_1_ background, TS-like cells could also be derived in CDM containing FAY (Table. 1), but these cells exhibited a rather differentiated cellular morphology and a slower proliferation rate (data not shown). Unexpectedly, in the 129×B6 F_1_ background, blastocysts grown in CDM containing 50 ng/ml FGF4, 20 ng/ml activin A, 10 nM XAV939, and 5 nM Y27632 did not gave rise to any TS cell lines (Table. 1). Often, epiblast stem cell (EpiSC) [15], [16]-like non-TS cells appeared in early passages of explants (Table. 1). These cells initially coexisted with TS cells, but because they proliferated more rapidly, they ultimately predominated over the TS cells (data not shown). As a control, we also established conventional TS cells from a pre-implantation (E3.5) 129×B6 F_1_ blastocyst. Furthermore, we were able to derive TS cell lines from extraembryonic ectoderm (ExE) dissected from a post-gastrulation (E6.5) 129×B6 F_1_ embryo cultured in CDM/FAXY (Fig. 1d, Table. 1).

**Table 1:** Establishment of TS cell lines in various conditions.

Number of embryos			Stage	Growth factors added to CDM				Lines derived		Rate (%)
CD1 × B6	CD1 × CD1	129 × B6		FGF2	Activin A	XAV939	Y27632	TS	Non-TS	
32			Blastocyst	+	+	+	+	18	10^a^	56
	12		Blastocyst	+	+	+	+	5	3^a^	42
		5	Blastocyst	+	+	+	+	5	0	100
16			Blastocyst		+	+	+	0	0	0
	12		Blastocyst		+	+	+	0	0	0
16			Blastocyst	+		+	+	0	0	0
	12		Blastocyst	+		+	+	0	0	0
16			Blastocyst	+	+		+	11^b^	5^a^	69
	12		Blastocyst	+	+		+	0	2^a^	0
16			Blastocyst	+	+	+		0	0	0
	12		Blastocyst	+	+	+		0	1	0
8			Blastocyst	+	+			0	1	0
	8		Blastocyst	+	+			0	0	0
8			Blastocyst		+	+		0	0	0
	8		Blastocyst		+	+		0	0	0
8			Blastocyst			+	+	0	0	0
	8		Blastocyst			+	+	0	0	0
8			Blastocyst	+			+	0	0	0
	8		Blastocyst	+			+	0	1	0
8			Blastocyst	+		+		0	0	0
	8		Blastocyst	+		+		0	0	0
8			Blastocyst		+		+	0	1	0
	8		Blastocyst		+		+	0	1	0
8			Blastocyst	+				0	0	0
	8		Blastocyst	+				0	0	0
8			Blastocyst		+			0	0	0
	8		Blastocyst		+			0	0	0
8			Blastocyst			+		0	0	0
	8		Blastocyst			+		0	0	0
8			Blastocyst				+	0	0	0
	8		Blastocyst				+	0	0	0
2			Morula	+	+	+	+	2	0	100
		6	E6.5 ExE	+	+	+	+	3	3^a^	50
16			Blastocyst	FGF4	+	+	+	0	1	0
10			Blastocyst	LIF + PD0325901 + CHIR99021				0	10^c^	0

a , EpiSC-like;

b , partially differentiated TSCs;

c , ESCs.

### Molecular properties of TS cells

Immunofluorescence staining of TS cells (129×B6 F_1_) after 22 passages revealed nuclear localization of caudal-type homeobox protein 2 (Cdx2), a trophoblast stem cell marker, in almost all cells (Fig. 1e). To compare the proliferation rates of the new and conventional TS cells, we seeded 2×10^5^ cells at each passage (i.e., at 2-day and 3-day intervals, respectively). The doubling time of new TS cells (approximately 9.2 hours) was remarkably shorter than that of conventional TS cells (approximately 24.1 hours) (Fig. 1f). Next, to compare the proliferation rates of the new TS and ES cells, we seeded 2×10^5^ cells at each passage (i.e., at 2-day intervals). The doubling time of the ES cells (approximately 8.9 hours) was slightly shorter than that of TS cells (Fig. 1g). The TS cells could be maintained for at least 30 passages (data not shown). To analyze the molecular state, we characterized and compared gene expression in TS and ES cells by quantitative PCR (qPCR). TS cells barely expressed ES cell–specific marker genes, including *Nanog*, *Cripro*, *Klf2*, *Oct3/4*, and *Klf4* (Fig. 1h). Conversely, they did express high levels of TS-cell marker genes, including eomesodermin (*Eomes*), E74-like factor 5 (*Elf5*), and *Cdx2*, whereas these genes were barely expressed in ES cells (Fig. 1h). Relative to ES cells, TS cells expressed higher levels of E-cadherin (*Cdh1*) and transcription factor activating enhancer-binding protein 2 gamma (*Tcfap2c*), and similar levels of Kruppel-like factor 5 (*Klf5*), retinoic acid–regulated zinc-finger gene (*Rex1*) [17], estrogen-related receptor beta (*Essrb*), SRY-related HMG-box 2 (*Sox2*), and T-box transcription factor 3 (*Tbx3*) (Fig. 1h). E3.5 blastocyst–derived TS cells and E6.5 ExE–derived TS cells exhibited remarkably similar expression patterns of these genes (Fig. 1i). Both TS and ES cells barely expressed ectodermal markers (*Nestin*, *Sox1*, *Ncam*), mesodermal markers (*Hoxa1*, *Hoxb1*, *Flk1*, *T*), and endodermal markers (*Gata4*, *Gata6*, *Afp*, *Foxa2*) (Fig. 1i).

**Figure 2:**
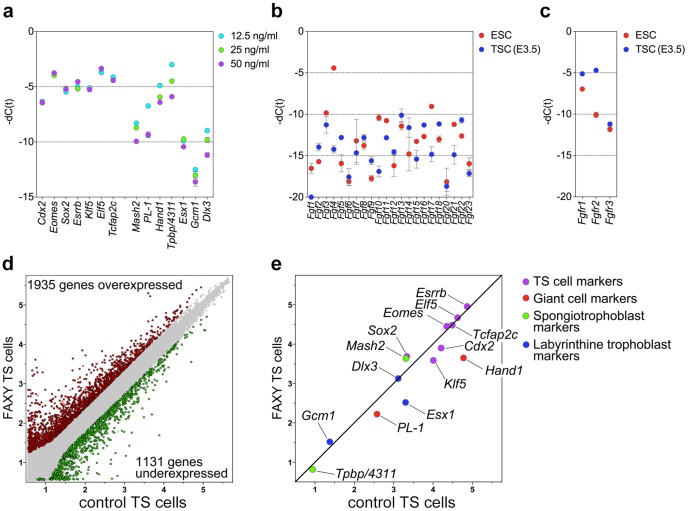
Gene-expression analyses of TS cells. (a) Dose-dependent effect of FGF2 on expression levels of TS cell marker genes and differentiated trophoblast lineage marker genes at 12.5 ng/ml (cyan), 25 ng/ml (green), and 50 ng/ml (magenta). (b) Comparison of expression levels of FGF ligand family genes between ES cells (red) and E3.5 blastocyst–derived TS cells (blue). (c) Comparison of expression levels of FGF receptor genes between ES cells (red) and E3.5 blastocyst–derived TS cells (blue). (d) Comparison of global gene expression analysis between conventional and new TS cells. (e) Highlighted expression levels of trophoblast stem-cell marker genes and differentiated trophoblast-subtype marker genes.

To assess the optimal concentration of FGF2 for maintaining TS cells in their undifferentiated state, we cultured E3.5 blastocyst–derived TS cells (129×B6 F_1_ background) in CDM/FAXY containing 12.5, 25, or 50 ng/ml FGF2. The concentration of FGF2 did not influence expression levels of TS cell marker genes (*Cdx2*, *Eomes*, *Sox2*, *Esrrb*, *Klf5*, *Elf5*, and *Tcfap2c*), but FGF2 did suppress expression of differentiated trophoblast lineage markers for trophoblast giant cells (placental lactogen 1 [*PL-1*] and *Homo sapiens* heart and neural crest derivatives expressed 1 [*Hand1*]), spongiotrophoblast cells (mammalian achaete-scute homologous protein 2 [*Mash2*] and trophoblast specific protein alpha [*Tpbp/4311*]), and labyrinthine trophoblasts (extraembryonic spermatogenesis homeobox 1 [*Esx1*], glial cells missing homolog 1 [*Gcm1*], and distal-less homeobox 3 [*Dlx3*]) in a dose-dependent manner (Fig. 2a).

We then analyzed and compared expression levels of FGF ligands in TS and ES cells. The FGF family consists of at least 23 members in vertebrates [18]. Only FGF4 was expressed at high levels in ES cells (Fig. 2b). We also analyzed expression levels of FGF receptor genes in TS and ES cells. TS cells expressed *Fgfr1* and *Fgfr2*, which is strongly expressed in diploid trophoblast [19], at high levels and *Fgfr3* at low levels (Fig. 2c). ES cells expressed only *Fgfr1* at high levels.

Next, we used microarray analysis to compare global gene expression between the new and conventional TS cells. In total, 3066 genes were differentially expressed by at least 2-fold. Among those 3066 genes, 1935 were overexpressed in the new TS cells, and 1131 genes were underexpressed (Fig. 2d, Table. S1). Both the new and conventional TS cells exhibited similar expression levels of trophoblast stem cell marker genes (*Cdx2*, *Eomes*, *Sox2*, *Esrrb*, *Klf5*, *Elf5*, and *Tcfap2c*) (Fig. 2e). Relative to conventional cells, the new TS cells expressed lower levels of *Hand1* and *PL-1* (giant cell marker genes) and *Esx1* (a labyrinthine trophoblast marker gene) (Fig. 2e).

### Requirement for FGF2, Activin A, and XAV939

**Figure 3:**
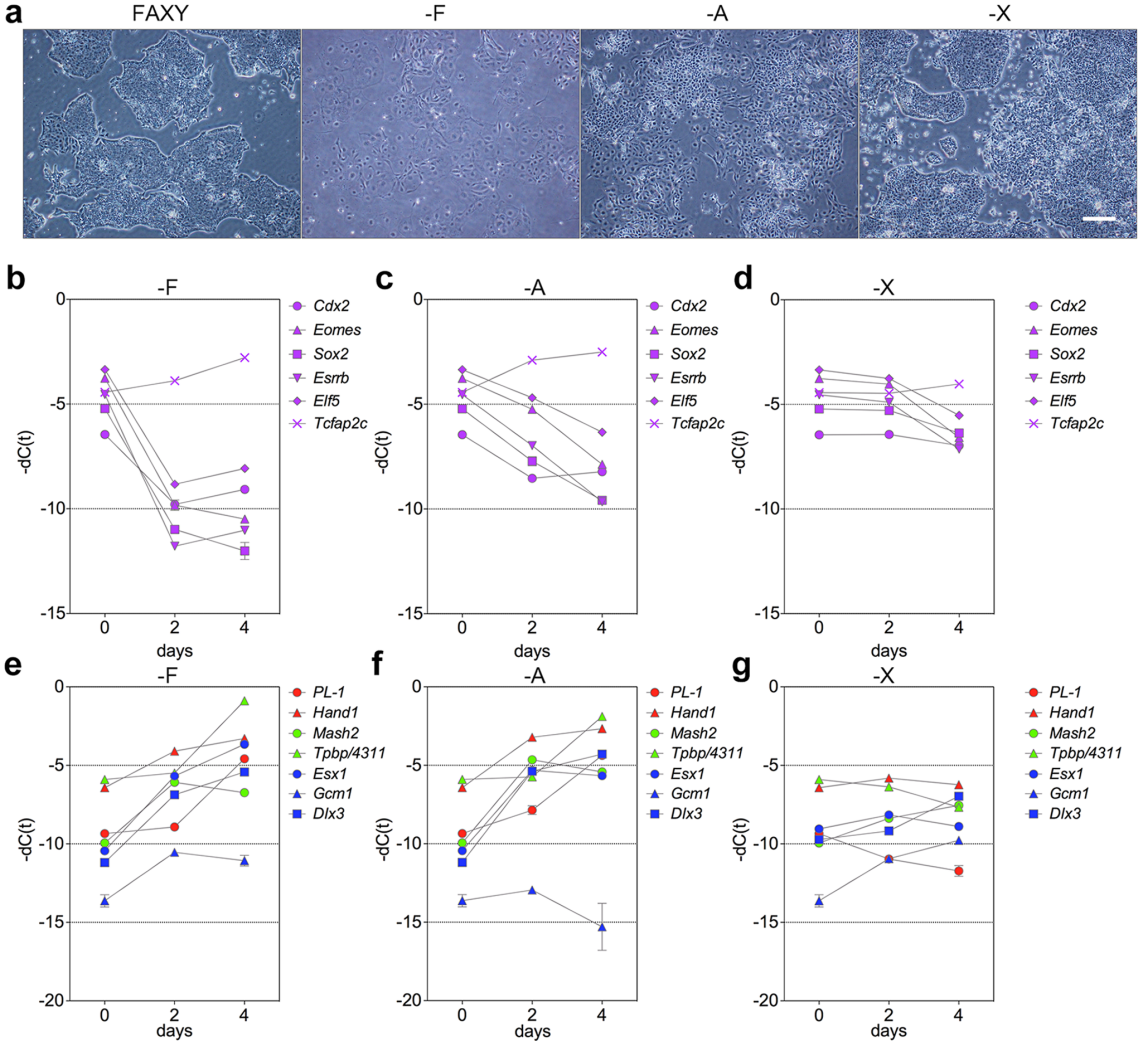
Differentiation capacity of TS cells *in vitro*. (a) Morphological changes of TS cells upon removal of FGF2 (-F, second from the left), activin A (-A, second from the right), or XAV939 (-X, right). FAXY represents undifferentiated TS cells as a control (left). Scale bar, 100 µm. (b) Changes in expression levels of TS cell marker genes upon removal of FGF2 (-F). (c) Changes in expression levels of TS cell marker genes upon removal of activin A (-A). (d) Changes in expression levels of TS cell marker genes upon removal of XAV939 (-X). (e) Changes in expression levels of differentiated trophoblast lineage marker genes upon removal of FGF2 (-F). (f) Changes in expression levels of differentiated trophoblast lineage marker genes upon removal of activin A (-A). (g) Changes in expression levels of differentiated trophoblast lineage marker genes upon removal of XAV939 (-X).

In order to determine which factors are required to maintain the tight stem cell–like colony morphology of the TS cells, we observed the morphological changes in TS cells resulting from removal of FGF2, activin A, or XAV939. For five passages prior to these experiments, the undifferentiated state of TS cells was maintained in CDM-FAXY (50 ng/ml FGF2). The removal of FGF2 or Activin A dramatically reduced the proliferation rate and induced differentiation, mainly into flat epithelial cells (Fig. 3a). The removal of XAV939 resulted in the consistent appearance of differentiated cells at the edges of colonies (Fig. 2a, Fig. S1).

Next, we characterized TS cells and their differentiated progeny by qPCR. In particular, we analyzed expression of markers for trophoblast stem cells (*Cdx2*, *Eomes*, *Sox2*, *Esrrb*, *Elf5*, *Tcfap2c*), trophoblast giant cells (*PL-1*, *Hand1*), spongiotrophoblast cells (*Mash2*, *Tpbp/-4311*), and labyrinthine trophoblasts (*Esx1*, *Gcm1*, *Dlx3*). The removal of FGF2 or activin A resulted in rapid downregulation of expression of TS-cell marker genes, with the exception of *Tcfap2c* (Fig. 3b, 3c), and a rapid upregulation of all trophoblast cell lineage markers with the exception of *Gcm1*, a labyrinthine trophoblast marker (Fig. 3e, 3f). The absence of XAV939 barely influenced the expression levels of *Cdx2* and *Tcfap2c*, but after 4 days, removal of this compound resulted in suppressed expression of *Eomes*, *Sox2*, *Esrrb*, and *Elf5* (Fig. 3d); upregulation of *Mash2*, *Dlx3*, and *Gcm1*; and downregulation of *PL-1* and *Tpbp/4311* (Fig. 3g).

### Requirement for Y27632

**Figure 4:**
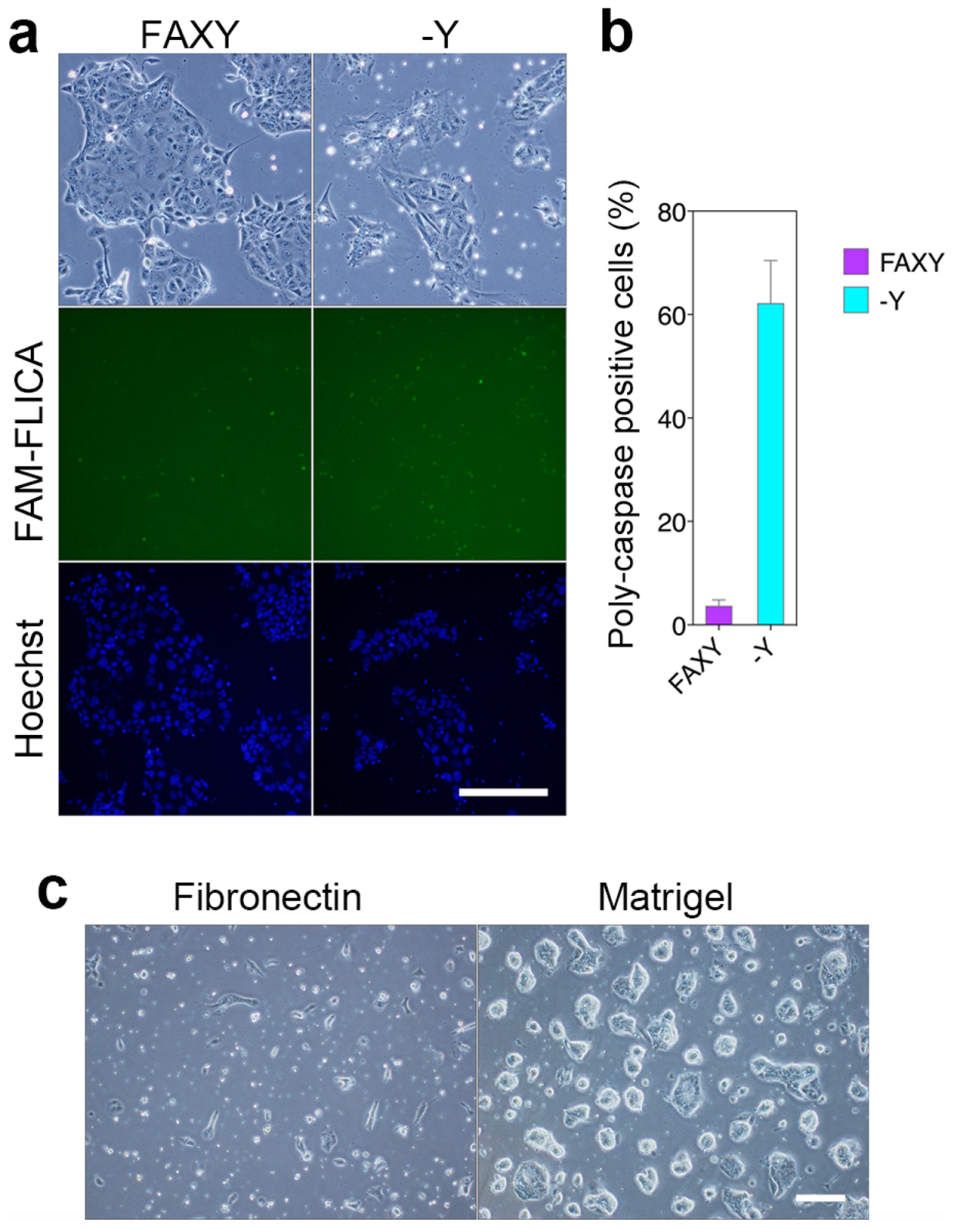
Requirement for Y27632. (a) Fluorescence-based detection of poly-caspase–positive cells by FAM-FLICA. Undifferentiated control (FAXY, left) and Y27632 removed (-Y, right). Scale bar, 100 µm. (b) Quantitation of poly-caspase–positive cells, expressed as a percentage (%) (c) Morphology of TS cell colonies on fibronectin (left) and Matrigel (right). Scale bar, 100 µm.

To verify the requirement for Y27632, we removed only Y27632 from cultures and investigated the effects. At 24 hours after the removal of Y27632, in contrast to the removal of FGF2, activin A, or XAV939, ∼60% of cells were poly-caspase–positive apoptotic cells, and very few cells survived (Fig. 4a, 4b). In addition, we screened for extracellular matrix that would allow TS cells could survive even after Y27632 removal. We found that the TS cells could be maintained on Matrigel-coated dishes for at least 20 passages in the absence of Y27632. These TS cells exhibited compact and dome-shaped colony morphology (Fig. 4c).

### Ability to contribute to placenta in chimeric mice

**Figure 5:**
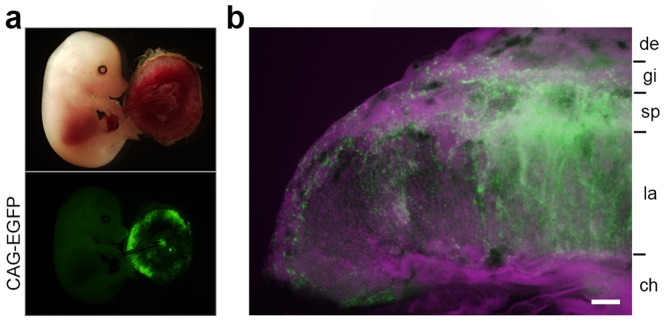
Differentiation capacity of TS cells *in vivo*. (a) Placenta-specific contribution of TS cells in an E14.5 embryo. (b) Merged image of DAPI staining (gray) and EGFP fluorescence (green) of a placental section at E14.5. de, decidua; gi, giant cells; sp, spongiotrophoblast cells; la, labyrinth; ch, chorion. Scale bar, 200 µm.

To analyze the ability of TS cells to contribute to placenta, we injected these cells into C57BL/6 blastocyst embryos (n = 100). To permit visualization of donor TS cells, the injected cells were first labeled with *CAG-EGFP* by lentivirus infection [20]. Donor TS cells contributed to the fetal portion of the placenta only at E14.5 (6/69, 8.7%)(Fig. 5a). TS cells differentiated into cells of all trophoblast subtypes: trophoblast giant cells, spongiotrophoblast cells, and labyrinthine trophoblasts (Fig. 5b). There were no TE-derived cells in the maternal decidua or extraembryonic mesodermal chorionic membrane (Fig. 5b).

## Discussion

Mouse ES cells can contribute to all types of embryonic cells in chimeras (including the germline) by transplantation into early embryos, and can generate almost entirely ES cell–derived individuals by tetraploid chimera formation [21]. However, the generation of individuals from ES cells without the use of oocytes or early embryos has not yet been reported. A reasonable explanation for this phenomenon is that ES cells cannot generate the trophoblast lineage. To date, however, aggregation of ES and TS cells has also failed to solve this problem. In order to move toward reconstruction of the early embryo from ES and TS cells, it is necessary to develop a defined platform for precise description and dissection of the molecular state of TS cells and ES–TS cell interactions.

In this study, we established new TS cell lines in the CDM/FAXY culture condition. In previous work, conventional TS cells have been derived and maintained on a feeder layer of primary mouse embryonic fibroblast (MEF) cells in the presence of FGF4, heparin, serum, and conditioned medium. This method allows the passage of colonies with a tight epithelial morphology. Removal of any one of the critical components of this system (FGF4, heparin, or the MEF feeder layer) quickly and significantly reduces the rate of cell division; after the cells cease proliferating, they differentiate into a state resembling giant cells [10]. Indeed, even when all components of the system are present, a small fraction of TS cells cultured in this manner differentiate into giant cells at the borders of colonies following passage [10]. Furthermore, the requirements for TS cell proliferation and maintenance are not precisely defined: because conditioned media from fibroblasts is necessary for long-term culture of TS cells in this system, it is likely that FGF4 is not the only factor required for trophoblast growth [10]. Recently, Kubaczka et al. reported derivation and maintenance of TS cells with chemically defined medium containing FGF4, heparin, and TGFβ1 on Matrigel [11]. However, this was not strictly a chemically defined culture condition, because Matrigel contains various undefined factors. On the other hand, new TS cells could be established and maintained on fibronectin-coated dishes in the absence of FGF4 in media containing FGF2, activin A, XAV939, and Y27632. This condition allowed the passage of colonies with typical TS cell–like epithelial morphology and stemness. Unexpectedly, CDM containing 50 ng/ml FGF4, 20 ng/ml activin A, 10 nM XAV939, and 5 nM Y27632 could not give rise to any TS cell lines (Table 1).

Over the past decades, molecular and genetic experiments in mice have shown that the main signaling pathway involved in the interaction between the trophoblast and ICM is the FGF pathway. The FGF family consists of at least 23 members in vertebrates, but only *Fgf4* was highly expressed in ES cells, and no Fgfs were highly expressed in the TS cells (Fig. 2b). Before implantation, *Fgf4* is widely expressed throughout the embryo. In the blastocyst, however, expression of *Fgf4* is limited to the ICM, consistent with a model in which FGF4 secreted by embryonic cells stimulates proliferation of the trophoblasts that will ultimately form the main structural components of the placenta [22]. In *Fgf4* mutant mice, embryos die at implantation, and both the ICM and trophoblast lineages exhibit developmental deficiencies [23]. Embryonic lethality in *Fgf4* mutants might result primarily from the inability of trophoblasts to develop into a functional placenta, as *Fgf4^−/−^* ES cells themselves are viable [24]. We compared expression levels of FGF receptors in TS and ES cells. TS cells expressed *Fgfr1* and *Fgfr2* at high levels, but barely expressed *Fgfr3* (Fig. 2c), whereas ES cells expressed only *Fgfr1* at high levels (Fig. 2c). *Fgfr2* expression levels in the diploid trophoblast are high in the blastocyst stage and immediately after implantation [19], consistent with the idea that FGFR2 is the critical receptor molecule in TS cells. As with *Fgf4* mutations, deficiencies in *Fgfr2* cause failures in placental development and embryonic lethality [25], [26]. These results suggest that although FGF4 and Fgfr2 are an ICM-derived ligand and receptor that support trophoblast proliferation *in vivo*, FGF4 cannot function as active ligand *in vitro* in the absence of a MEF feeder layer, FBS, and conditioned medium. FGF2, however, could act as an active FGF ligand, allowing derivation and maintenance of the TS cells in the absence of the undefined factors.

When we derived the TS cell lines in CDM/FAXY, epiblast stem cell (EpiSC) [15], [16]-like non-TS cells often appeared in early passages of explants (Table. 1). The CDM-FAXY condition resembles a modified EpiSC culture condition [27], which can support the undifferentiated state of the EpiSCs closely other than their basal media. Therefore, if epiblast-like cells emerge from ICM-derived outgrowths and epiblast cells adjacent to the ExE contaminate the primary cultures used for TS cell derivation, CDM-FAXY may be able to give rise to EpiSCs.

The new and conventional TS cells exhibited similar expression levels of trophoblast stem cell marker genes (*Cdx2*, *Eomes*, *Sox2*, *Esrrb*, *Klf5*, *Elf5*, and *Tcfap2c*) (Fig. 2e). Relative to conventional cells, new TS cells expressed lower levels of giant cell marker genes, *Hand1* and *PL-1*, and a labyrinthine trophoblast marker gene, *Esx1* (Fig. 2e). The removal of FGF4 from conventional TS culture conditions (i.e., containing undefined serum factors and feeder cells) results in subsequent differentiation into cells with a giant cell–like phenotype [10]. By contrast, removal of FGF2 or activin A from CDM/FAXY resulted in a rapid decline in proliferation, with subsequent differentiation into cells of all trophoblast cell lineages: trophoblast giant cells, spongiotrophoblast cells, and labyrinthine trophoblasts (Fig. 3a, b, c, e, f). This unbiased differentiation potential suggests that the new TS cells retain their undifferentiated state *in vitro*. The removal of XAV939 resulted in gradual differentiation, down-regulation of some TS cell marker genes, and alterations in the expression levels of differentiated trophoblast lineage marker genes (Fig. 3a, 3d, 3g). These results suggested that XAV939 is necessary to stably maintain the undifferentiated state of TS cells, and that this compound acts via a pathway distinct from those FGF2 and activin A. The requirement of Y27632 for TS cell survival could be circumvented by culturing the cells on Matrigel-coated dishes, although the resultant cells had colony morphologies distinct from those of typical TS cells (Fig. 4c). This result suggests two possibilities: either Matrigel provides extracellular matrix that allows TS cells to survive independent of Y27632, or Matrigel contains undefined factors that satisfy the Y27632 dependency.

Interference with several signaling pathways promotes the pluripotency of ES cells: suppression of Fgf signaling by inhibition of Fgfr, mitogen-activated protein kinase kinase (Mek), and/or mitogen-activated protein kinase (Mapk); inhibition of Tgf-β/activin signaling by the inhibition of their type I receptors, activin receptor-like kinase 4 (Alk4), Alk5, and Alk7; and activation of Wnt signaling by the inhibition of glycogen synthase kinase 3 (Gsk3) [8], [9], [28]. By contrast, our results show that activation of Fgf signaling by FGF2, activation of activin signaling by activin A, and suppression of Wnt signaling by XAV939 allow derivation and maintenance of TS cells in chemically defined condition. Thus, ES cells and TS cells, which respectively reflect the early embryonic properties of ICM and TE, are derived and maintained under dissimilar signaling conditions.

The TS cells we derived using CDM/FAXY could contribute to all trophoblast subtypes: trophoblast giant cells (gi), spongiotrophoblast cells (sp), and labyrinthine trophoblasts (la) (Fig. 5a, 5b). This unbiased differentiation potential, together with the trophoblast lineage–specific contribution *in vivo* in chimeras, suggests that TS cells retain their undifferentiated state.

Our results show that the TS cells satisfied all the criteria for undifferentiated trophoblast stem cells: self-renewal capacity, marker gene expression, differentiation competence into various trophoblast lineage cells *in vitro*, and the ability to contribute to the placenta in chimeras. Defining the signaling pathways that maintain TS cells may provide key insights into early trophoblast development, elucidate the molecular interactions between TE and ICM, and facilitate the development of technologies using TS and ES cells.

## Materials and Methods

### TS cell derivation

The following condition, termed CDM/FAXY, was used to derive and maintain undifferentiated TS cells from pre-implantation E3.5 blastocysts and post-implantation E6.5 extraembryonic ectoderms. CDM/FAXY was generated by combining 250 ml of Neurobasal (Invitrogen), 250 ml of DMEM/F12 (Ham's) (1∶1) (Invitrogen), 2.5 ml N2 supplement (Invitrogen), B27 supplement (Invitrogen), 5 ml penicillin–streptomycin–L-glutamine solution (Invitrogen), bovine serum albumin (Sigma, 0.05% final concentration), 1-thioglycerol (Sigma, 1.5×10^−4^ M final concentration), recombinant mouse basic FGF (R&D, 12.5–50 ng/ml final concentration), recombinant human activin A (R&D, 20 ng/ml final concentration), XAV939 (Calbiochem, 10 nM final concentration), and Y27632 (Stemgent, 5 nM/ml final concentration). E3.5 blastocysts and E6.5 extraembryonic ectoderms were placed on 96-well plates coated with 15 µg/ml human plasma fibronectin (Millipore) for at least 1 hour at 37°C in CDM/FAXY. Initial outgrowths of proliferating TS cells were evident by day 5; these outgrowths were dissociated into single cells using TripLE Select (Invitrogen) 5–8 days following plating. The newly established cell lines were further propagated on fibronectin-coated dishes by TripLE Select and then either frozen using a Cellbanker 2 (Mitsubishi Chemical Medience) or used for further analysis. Medium was replaced every 2 days. KnockOut serum replacement (Invitrogen, 1% final concentration) can be used in CDM/FAXY and result in positive cell adherent effect (data not shown). Dishes coated with 0.2% Matrigel (BD Biosciences) were used for TS cell culture when Y27632 was not added. Conventional TS cells (129B6F1) were derived and maintained with FGF4, heparin, and serum containing MEF-conditioned medium, as previously described [10].

### Quantitative PCR (qPCR) analysis

Total RNA was purified from cells using RNeasy Mini kits (Qiagen). For reverse transcription, ReverTra Ace (Toyobo) and oligo (dT)_20_ primer were used. For qPCR PCR analyses, Power SYBR Green PCR Master Mix (Applied Biosystems) and a CFX384 Real-Time System (Bio-Rad) were used. Transcript levels were determined in triplicate reactions and normalized against the corresponding levels of *Gapdh*. Primer sequences are shown in Table S2.

### Microarray analysis

Total RNA was purified by using RNeasy Mini kit (QIAGEN). Microarray targets from 200 ng total RNA were synthesized and labeled using the Low RNA Input Linear Amp Kit (Agilent) and hybridized to Mouse 4×44K Ver.2.0 arrays. Arrays were scanned on an Agilent Technologies Microarray scanner, and signal intensities were calculated using the Agilent Feature Extraction 10.7.3.1 software. GEO accession number is GSE59107.

### Immunofluorescence staining

Cells were grown for 2 days on a fibronectin-coated plastic-bottom dish (35 mm µ-Dish; *ibid*.), fixed with 4% paraformaldehyde/phosphate buffer for 1 hour at room temperature, and then washed three times with 0.1% Triton X100/PBS. Cells were blocked with blocking solution (0.5% normal goat serum [Vector] in PBS +0.1% Triton X-100 [PBST]) and incubated overnight at 4°C with primary antibody diluted in blocking solution. Cells were then washed three times with PBST, incubated with Alexa Fluor 568–conjugated goat anti–rabbit IgG antibody (1∶500; A11036, Invitrogen) for 1 hour at room temperature, washed in PBST, counterstained with DAPI (1 µg/ml; Invitrogen), and imaged. The rabbit anti-Cdx2 antibody (ab76541, Abcam) was used at 1∶500 dilution.

### Apoptosis analysis

Apoptotic cells were detected using the FAM-FLICA Poly Caspase Assay Kit (ImmunoChemistry Technologies), and cells were counterstained with Hoechst 33342 (200 µg/ml, ImmunoChemistry Technologies).

### Imaging

Images were acquired with an IX71 inverted microscope (Olympus) equipped with a DP72 camera (Olympus) or with an SZX12 dissection microscope (Olympus) equipped with a DP71 camera (Olympus).

### Lentivirus production and infection

293FT cells were plated at 4×10^6^ cells per 100 mm dish and incubated overnight. Cells were then transfected with the pLV-CAG1.1-EGFP, pMDL g/p, pREV, and pVSVG vectors [20] using Lipofectamine 2000 (Invitrogen). At 48 and 72 hours after transfection, medium from the transfectants was collected and filtered through a 0.45- µm pore cellulose acetate filter (Millipore); the filtrate was used as the viral supernatant. One day before transduction, TS cells were seeded at 1×10^5^ cells per well in 6-well plates (Greiner Bio One). On the day of transduction, the medium was replaced with viral supernatant supplemented with 4 mg/ml Polybrene (Nacalai Tesque), and then incubated for 24 hours.

### Blastocyst injection

To generate chimeric mice, three to five TSCs were injected into C57BL/6 blastocysts, which were then transferred into the uterine horns of CD-1 pseudopregnant mice.

### Animal ethics statement

All animal experiments conformed to our guidelines for the care and use of laboratory animals and were approved by the institutional committee for laboratory animal experimentation (RIKEN Kobe Institute). CO_2_ inhalation was used for euthanasia. To ensure death following CO_2_ asphyxiation, cervical dislocation was performed.

## Supporting Information

Figure S1 **Morphological changes of TS cells upon removal of XAV939 (-X, right) at higher magnification.** FAXY represents undifferentiated TS cells as a control (left). Scale bar, 100 µm.
(JPG)

Table S1 **Global comparison of gene expression between conventional TS cells and new TS cells.**
(XLSX)

Table S2 **Sequences of oligonucleotide primers.**
(XLS)
