# Correction: Establishment of Trophoblast Stem Cells under Defined Culture Conditions in Mice

**DOI:** 10.1371/journal.pone.0121167

**Published:** 2015-03-20

**Authors:** The PLOS ONE Staff

There is an error in the third sentence of the first paragraph of the “Establishment and self-renewal of TS cells in chemically defined conditions” subsection of the Results. The correct sentence is: To this end, we cultured CD1 × B6 F_1_ GOF18 blastocysts harboring the *Oct3/4-EGFP* reporter (Fig. 1a) [14] in CDM containing LIF, PD0325901 (a MEK inhibitor), and CHIR99021 (a GSK3 inhibitor) (CDM/L2i, the ES cell culture condition) or CDM containing FAXY (12.5 ng/ml FGF2, 20 ng/ml activin A, 10 μM XAV939, 5 μM Y27632) (CDM/FAXY, the TS cell culture condition).

There is an error in the fifth to last sentence of the first paragraph of the “Establishment and self-renewal of TS cells in chemically defined conditions” subsection of the Results. The correct sentence is: Unexpectedly, in the 129×B6 F_1_background, blastocysts grown in CDM containing 50 ng/ml FGF4, 20 ng/ml activin A, 10 μM XAV939, and 5 μM Y27632 did not gave rise to any TS cell lines (Table. 1).

There is an error in the last sentence of the second paragraph of the Discussion. The correct sentence is: Unexpectedly, CDM containing 50 ng/ml FGF4, 20 ng/ml activin A, 10 μM XAV939, and 5 μM Y27632 could not give rise to any TS cell lines (Table 1).

There is an error in the second sentence of the “TS cell derivation” subsection of the Materials and Methods. The correct sentence is: CDM/FAXY was generated by combining 250 ml of Neurobasal (Invitrogen), 250 ml of DMEM/F12 (Ham's) (1:1) (Invitrogen), 2.5 ml N2 supplement (Invitrogen), B27 supplement (Invitrogen), 5 ml penicillin–streptomycin–L-glutamine solution (Invitrogen), bovine serum albumin (Sigma, 0.05% final concentration), 1-thioglycerol (Sigma, 1.5×10^−4^ M final concentration), recombinant mouse basic FGF (R&D, 12.5–50 ng/ml final concentration), recombinant human activin A (R&D, 20 ng/ml final concentration), XAV939 (Calbiochem, 10 μM final concentration), and Y27632 (Stemgent, 5 μM/ml final concentration).
