# Supplementary figures and images for: Establishment of Trophoblast Stem Cells under Defined Culture Conditions in Mice
Source: PLoS One. 2014 Sep 9;9(9):e107308. doi: 10.1371/journal.pone.0107308 (PMC4159327; doi:10.1371/journal.pone.0107308)

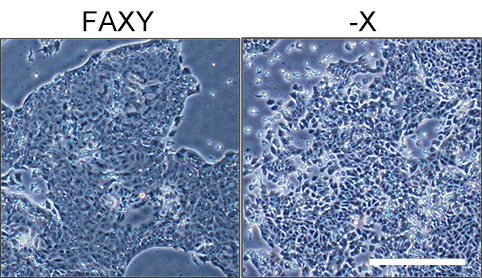

Supplement: Figure S1 — Morphological changes of TS cells upon removal of XAV939 (-X, right) at higher magnification. FAXY represents undifferentiated TS cells as a control (left). Scale bar, 100 µm. (JPG) [file pone.0107308.s001.jpg]
